# The Pervasive Effects of ER Stress on a Typical Endocrine Cell: Dedifferentiation, Mesenchymal Shift and Antioxidant Response in the Thyrocyte

**DOI:** 10.3389/fendo.2020.588685

**Published:** 2020-11-09

**Authors:** Luca Ulianich, Paola Mirra, Corrado Garbi, Gaetano Calì, Domenico Conza, Antonella Sonia Treglia, Alessandro Miraglia, Dario Punzi, Claudia Miele, Gregory Alexander Raciti, Francesco Beguinot, Eduardo Consiglio, Bruno Di Jeso

**Affiliations:** ^1^ Dipartimento di Scienze Mediche e Traslazionali Universita’ “Federico II” & URT dell’Istituto di Endocrinologia e Oncologia Sperimentale “Gaetano Salvatore,” Consiglio Nazionale delle Ricerche (CNR), Napoli, Italy; ^2^ Dipartimento di Medicina Molecolare e Biotecnologie Mediche, Napoli, Italy; ^3^ Istituto di Endocrinologia ed Oncologia Sperimentale “G. Salvatore,” CNR, Napoli, Italy; ^4^ Dipartimento di Scienze e Tecnologie Biologiche ed Ambientali, Università del Salento, Lecce, Italy

**Keywords:** ER stress, thyroid, dedifferentiation, mesenchymal phenotype, antioxidant response

## Abstract

The endoplasmic reticulum stress and the unfolded protein response are triggered following an imbalance between protein load and protein folding. Until recently, two possible outcomes of the unfolded protein response have been considered: life or death. We sought to substantiate a third alternative, dedifferentiation, mesenchymal shift, and activation of the antioxidant response by using typical endocrine cells, i.e. thyroid cells. The thyroid is a unique system both of endoplasmic reticulum stress (a single protein, thyroglobulin represents the majority of proteins synthesized in the endoplasmic reticulum by the thyrocyte) and of polarized epithelium (the single layer of thyrocytes delimiting the follicle). Following endoplasmic reticulum stress, in thyroid cells the folding of thyroglobulin was disrupted. The mRNAs of unfolded protein response were induced or spliced (X-box binding protein-1). Differentiation was inhibited: mRNA levels of thyroid specific genes, and of thyroid transcription factors were dramatically downregulated, at least in part, transcriptionally. The dedifferentiating response was accompanied by an upregulation of mRNAs of antioxidant genes. Moreover, cadherin-1, and the thyroid (and kidney)-specific cadherin-16 mRNAs were downregulated, vimentin, and SNAI1 mRNAs were upregulated. In addition, loss of cortical actin and stress fibers formation were observed. Together, these data indicate that ER stress in thyroid cells induces dedifferentiation, loss of epithelial organization, shift towards a mesenchymal phenotype, and activation of the antioxidant response, highlighting, at the same time, a new and wide strategy to achieve survival following ER stress, and, as a sort of the other side of the coin, a possible new molecular mechanism of decline/loss of function leading to a deficit of thyroid hormones formation.

## Introduction

The endoplasmic reticulum (ER) is the cellular organelle where newly synthesized secretory and transmembrane (cargo) proteins are cotranslationally translocated and folded. Only correctly folded proteins can move on along the secretory pathway, while misfolded proteins are retained in the ER and eventually degraded trough endoplasmic reticulum-associated degradation (ERAD) (1). Protein misfolding may arise when the ER environment is perturbed by, among others, alteration of calcium homeostasis or redox status, increased cargo protein synthesis, or/and altered glycosylation, placing a condition of stress on the ER.

When ER stress ensues, an adaptive mechanism, the unfolded protein response (UPR) is triggered. The UPR involves transcriptional induction of genes that enhance ER protein folding capacity and promote ERAD (1). Translation of mRNAs is also initially inhibited, together with cotranslational degradation of secretory proteins and degradation of ER-localized mRNAs (1). However, when ER stress is excessive or prolonged and recovery fails, the UPR activates an apoptotic program (1). Indeed, much attention has been devoted to the understanding of the life/death switch mechanism (2–4). However, recent reports have contributed to the idea that adaptation does not necessarily mean full recovery of the pre-existing function. Indeed, reprogramming gene expression to a less differentiated state after ER stress has been shown in a number of systems (5–11).

We sought to extend the concept of regression of differentiation to tissue organization, particularly on endocrine cells. We reasoned that the thyroid may represent an ideal system to this aim, since the thyrocyte is a typical endocrine cell. Thus, the thyrocyte must synthesize much more of a single protein [thyroglobulin (Tg), which accounts for about 50% of newly synthesized cargo proteins of the thyrocyte] than any other protein (12–19) such like, for example, pancreatic β-cells (20), and, indeed, both cell type are particularly susceptible to ER stress (14, 20). In addition, endocrine function is often related to a complex tissue structural organization. Thus, thyroid function is based on the follicle, a single layer of polarized thyrocytes delimiting a central cavity of the follicle (19), and, for example, the function of the pancreatic β-cell, is based on the complex structural organization of the pancreatic islet (21). To test these two different aspects of regression to a less differentiated state, we decided to use two thyroid cell lines, the fully differentiated thyroid cell line PCCl3 (22), and the highly polarized FRT thyroid cell line (23).

## Materials and Methods

### Cell Culture and Th/Tn Treatments

PCCl3 and FRT cells were cultured as previously reported (12, 22, 23). In brief, these cells were grown in Coon’s modified Ham’s F-12 medium supplemented with 5% calf serum and a mixture of six hormones and growth factors, i.e. insulin (1 μg/ml), TSH (1 mIU/ml), cortisone (10 nM), human transferrin (5 pg/ml), somatostatin (10 ng/ml), and glycyl-histidyl-L-lysine (10 ng/ml) (referred as complete medium). Thapsigargin (Th) and tunicamycin (Tn) (Calbiochem Merck) were added to the medium for 30 min, followed by 24 h in fresh complete medium without Th/Tn.

### Plasmids and Antibodies

The luciferase reporter plasmid paired box gene 8 (Pax8LUC) was provided by Dr. P.A. Kopp. Antibodies were directed towards: Tg (12), β-actin, tubulin, SNAI1, vinculin (Santa Cruz Biotechnology), cadherin-1 (CDH1) (Cell Signaling Technology Inc.), thyroid (and kidney)-specific cadherin-16 (CDH16) (provided by Dr. G. Calì), activating transcription factor-4 (ATF4) (Cell Signaling, Danvers, MA, USA), phospho-eukaryotic translation initiation factor 2 alpha (p-eIF2α) (Abnova, Taipei, Taiwan). Horseradish peroxidase-conjugated anti-mouse and anti-rabbit antibodies were from Amersham Biosciences.

### Cell Viability Assay

The conversion of MTT (3-(4,5-dimethylthiazol-2-yl)- 2,5-diphenol tetrazolium bromide) by PC Cl3 cells was used as an indicator of cell number as described by Mosmann (24). PC Cl3 cells were grown in 35 mm diameter plates for 48 h in complete medium. Th/Tn were added to the medium for 30 min, followed by 24 h in fresh complete medium without Th/Tn. MTT (0.5 mg/ml) was added to the cells for a 4-h incubation and cells were lysed in acidified isopropanol/HCl 0.04N. The lysates were subsequently read on a spectrophotometer at 550 nm (Bio-rad, Richmond, CA, USA) after a 1:2 dilution with water. The results were expressed as percent viability compared to control.

### RNA Isolation and Real-Time Reverse Transcription-Polymerase Chain Reaction (RT-PCR)

Total RNA was extracted with the TRIzol reagent, according to the manufacturer’s protocol. Reverse transcription of 1 μg of total RNA was performed using SuperScript III, following the manufacturer’s instructions. Quantitative real-time RT-PCR analysis was performed as previously described (25). Briefly, reactions were performed in triplicate by using iQ SYBR Green Supermix on iCycler real time detection system (Bio-Rad). Relative quantification of gene expression was calculated by the ΔΔ*Ct* method. Each *Ct* value was first normalized to the respective *Glyceraldehyde-3-Phosphate Dehydrogenase (GAPDH) Ct* value of a sample to account for variability in the concentration of RNA and in the conversion efficiency of the RT reaction. GAPDH was not affected by Th/Tn treatments. The primers used are listed in **Supplementary Materials** (**Table S1**).

### Immunofluorescence

1.5 × 10^5^ cells were plated on 12 mm diameter glass coverslips. Forty-eight hours later, cells were vehicle-treated or treated plus 0.5 μg/ml Tn or 0.5 μM Th for 30 min. The medium was then replaced with medium without Th/Tn and cells incubated for 24 h. Immunofluorescence was performed as previously reported (26). Briefly, cells were fixed in 4% paraformaldehyde in PBS for 20 min, washed twice in 50 mm NH_4_Cl in PBS, and permeabilized for 5 min in 0.1% Triton X-100 in PBS. Nuclei were stained with HOECHST 33258. Immunofluorescence analysis was performed at a confocal laser scanning microscope LSM 510 Meta (Zeiss, Gottingen, Germany). The λ of diode UV laser was 405, the argon ion laser was set at 488 nm. Fluorescence emission was revealed by 420–480 band pass filter for Hoechst and by 505–530 band pass filter for Alexa Fluor 488. Double staining IF images were acquired separately in the green, and blue channels at a resolution of 1,024 × 1,024 pixels, with the confocal pinhole set to one Airy unit and then saved in TIFF format.

### Transient Expression Analysis

Cells were plated in six-well plates to about 80% confluence 24 h before transfection. Cells were washed with serum-free medium before addition of 1 ml of plasmid/Lipofectamine mixture. The plasmid/Lipofectamine mixture was made by incubating 2.5 μg of luciferase reporter plasmid and 0.5 μg of pRL-TK vector (Promega) with 5 µl Lipofectamine 2000 (Invitrogen) and 200 µl of serum-free medium for 30 min at room temperature, before dilution with 800 µl serum-free medium. Cells were incubated for 5 h at 37°C before addition of 1 ml medium supplemented with 20% serum. After 24 h, cells were treated with 0.5 and 1.0 μg/ml of Tn for 30 min, 1 h, and 2 h. The medium was then replaced with medium without Tn. Twenty-four hours later, firefly and renilla activities were determined in cell lysates using the Dual-Luciferase Reporter Assay System (Promega) and a luminometer (Orion I, Berthold Detection Systems) according to the manufacturer’s instructions. Results were expressed as the ratio of firefly to renilla activity.

### Western Blots Analysis

Western blots were carried out as previously reported (16). Briefly, cells were treated or mock treated with Th or Tn in medium for 30 min, followed by 24 h in medium without Th/Tn. After evaluation of protein content, 30 μg of cell extract was analyzed by SDS-PAGE and electrotransferred to polyvinylidene difluoride. Blocking was for 15 h at 4°C with Tris-buffered saline-Tween 20 (TBST) buffer (10 mM Tris [pH 8.0], 150 mM NaCl, 0.1% Tween 20) containing 10% nonfat dry milk, followed by incubation in TBST buffer for 2 h at room temperature with a 1:2,000 dilution of anti-Tg, 1:500 anti-p-eIF2α, 1:1,000 anti-ATF4/antiCDH1/antiCDH16/antiSNAI1/anti vinculin, 1:2,000 anti-β-actin/anti-tubulin. After being washed with TBST, the blot was incubated for 1 h at room temperature with antirabbit horseradish peroxidase-conjugated antibodies diluted 1:3,000 in TBST. Band detection was by enhanced chemiluminescence. The molecular mass markers were from Euroclone.

### Statistical Procedures

Data are presented as means ± SD of at least three independent experiments, each performed in triplicate. The difference between groups was evaluated using Student’s t test. p < 0.05 was considered significant. *p < 0.05, **p < 0.01, and ***p < 0.001.

## Results

### Th/Tn Cause Retention of Tg in the ER and Activate the UPR

**Figure 1:**
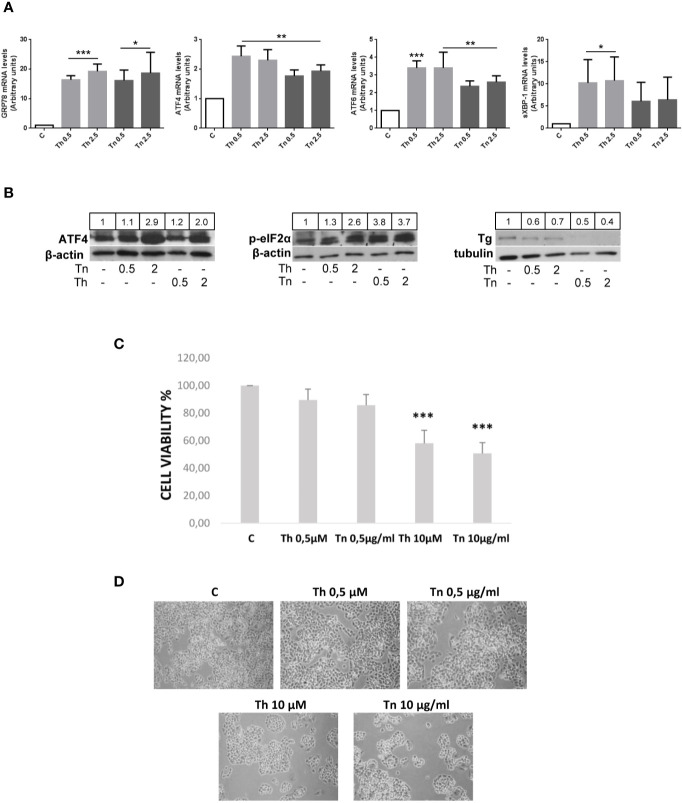
Th/Tn induce ER stress and UPR activation in PCCl3 cells without appreciably affecting viability. **(A)** Cells were plated in 100 mm diameter plates to about 80% confluence 24 h before treatments. Cells were treated or mock treated for 30 min with various concentrations of Th/Tn, followed by 24 h in complete medium without Th/Tn. Total RNA was extracted with the TRIzol reagent, according to the manufacturer’s protocol. Quantitative real-time RT-PCR analysis was performed as described in *Materials and Methods*. PCCl3 cells vehicle-treated (C) or treated with increasing concentrations of Th/Tn. *p < 0.05, **p < 0.01, ***p < 0.001, of each group respect to control. **(B)** Cells were plated in 60 mm diameter plates to about 80% confluence 24 h before treatments. Cells were treated or mock treated for 30 min with various concentrations of Th/Tn, followed by 24 h in complete medium without Th/Tn. Western blots of total protein extracts from PCCl3 cells vehicle-treated or treated with increasing concentrations of Th/Tn. The ratio of the densitometric values ATF4/β-actin, p-eIF2-α/β-actin, and Tg/tubulin is reported. **(C, D)** Cells were plated in 35 mm diameter plates to about 50% confluence 48 h before treatments. Cells were treated or mock treated for 30 min with various concentrations of Th/Tn, followed by 24 h in complete medium without Th/Tn. Cells were photographed by a Nikon Eclipse TS100 inverted microscope. Successively, MTT (0.5 µg/ml) was added to the cells for a 4-h incubation and cells were lysed in acidified isopropanol/HCl 0.04N. The lysates were subsequently read on a spectrophotometer at 550 nm (Bio-rad, Richmond, CA, USA) after a 1:2 dilution with water. The results were expressed as percent viability compared to controls. ***p < 0.001, of each group respect to control.

The widely used ER stress inducing agents Th and Tn induce in thyroid cells misfolding of Tg, its retention in the ER, and activation of the UPR (13–16). As shown in **Figure 1A**, PCCl3 cells treated for 30 min with various concentrations of Th/Tn, followed by 24 h in complete medium without Th/Tn, increased glucose-regulated protein 78 (GRP78), ATF4, activating transcription factor-6 (ATF6), and spliced active form of X-box binding protein-1 (XBP-1s) mRNA, even at the lowest concentration investigated (**Figure 1A**). The activation of the UPR was confirmed at the protein level, by increased ATF4 and phospho-eIF2α (**Figure 1B**, and **Supplementary Figures S1**, **S2**, **S3**, **S4**, **S5**, **S9**). Of note, our specific treatment protocol (30 min treatment with relatively low doses of Th/Tn, 0.5 µM and 0.5 µg/ml, respectively, followed by removal of the drug and incubation in complete medium) allowed us to substantially avoid cell death, as shown by cell viability assay and light microscopy imaging (**Figures 1C, D**). Instead, cell death occurred with doses of Th/Tn twenty-fold greater (**Figures 1C, D**).

### ER Stress Results in Decreased Thyroid-Specific Gene Expression and Activation of an Antioxidant Response in PCCl3 Cells

**Figure 2:**
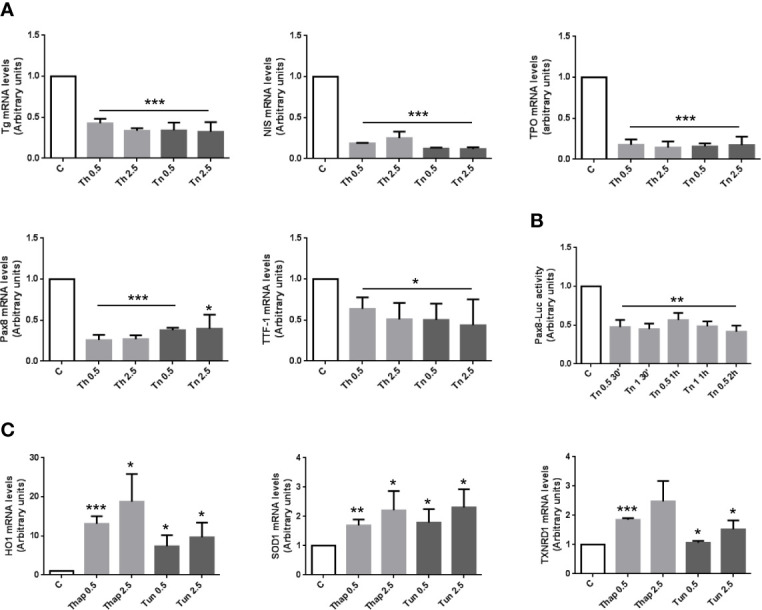
ER stress induces an inhibition of differentiation with a mechanism, at least in part, transcriptional, and an antioxidant response in PCCl3 cells. **(A)** Cells were plated in 100 mm diameter plates to about 80% confluence 24 h before treatments. Cells were treated or mock treated for 30 min with various concentrations of Th/Tn, followed by 24 h in complete medium without Th/Tn. Total RNA was extracted with the TRIzol reagent, according to the manufacturer’s protocol. Quantitative real-time RT-PCR analysis was performed as described in *Materials and Methods*. PCCl3 cells vehicle-treated (C) or treated with increasing concentrations of Th/Tn. *p < 0.05, ***p < 0.001, of each group respect to control. **(B)** Cells were plated in six-well plates to about 80% confluence 24 h before transfection. PCCl3 cells transfected with 2.5 μg of luciferase reporter plasmid and 0.5 μg of pRL-TK vector with 5 µl Lipofectamine 2000, as reported in *Materials and Methods*. Twenty-four hours after transfection cells were vehicle-treated or treated with 0.5 or 1.0 μg/ml Tn for 30, 60, and 120 min and harvested after 24 h in medium without Tn. Firefly and renilla activities were determined in cell lysates using the Dual-Luciferase Reporter Assay System and a luminometer. Results were expressed as the ratio of firefly to renilla activity. **p < 0.01, of each group respect to control. **(C)** Cells were plated in 100 mm diameter plates to about 80% confluence 24 h before treatments. Cells were treated or mock treated for 30 min with various concentrations of Th/Tn, followed by 24 h in complete medium without Th/Tn. Total RNA was extracted with the TRIzol reagent, according to the manufacturer’s protocol. Quantitative real-time RT-PCR analysis was performed as described in *Materials and Methods*. PCCl3 cells vehicle-treated (C) or treated with increasing concentrations of Th/Tn. *p < 0.05, **p < 0.01, ***p < 0.001, of each group respect to control.

To study if and how the expression of thyroid-specific genes was affected by an ER stress, we treated PCCl3 cells with Th/Tn following the same protocol of **Figure 1**. Th/Tn, even at the lowest doses, dramatically decreased mRNAs of thyroid-specific markers, Tg, sodium-iodide symporter (NIS), and thyroperoxidase (TPO) (**Figure 2A**). Transcription of Tg, TPO and NIS genes is directed by a combination of thyroid-specific transcription factors, mostly thyroid transcription factor 1 (TTF-1) and Pax-8, with Pax-8 playing a critical role (27). Th/Tn decreased the mRNAs levels of TTF-1 and Pax-8 (**Figure 2A**). Extending these results, Tg protein levels, in total extracts from Th/Tn-treated PC Cl3 cells, exhibited a dramatic decrease (**Figure 1B**). These results suggested that decreased Pax-8 transcriptionally caused a downregulation of Tg, TPO, and NIS genes. However, a more subtle question was as to whether the downregulation of the transcription factor itself had a transcriptional component. This was, in fact, the case, as shown by Pax8 promoter-luciferase assay (**Figure 2B**). These data indicate that ER stress induced by Th/Tn inhibits thyroid-specific gene expression, at least in part, transcriptionally in PCCl3 cells.

Next, we reasoned that the dedifferentiating response may not be the only one executed by thyroid cells in light of an adaptive effort to ER stress. Thus, ER stress activated also an antioxidant response, as shown by the increase in mRNA levels of heme oxigenase 1 (HO1), superoxide dismutase 1 (SOD1), and thioredoxin reductase 1 (TXNRD1) (**Figure 2C**).

### ER Stress Induces a Shift Towards a Mesenchymal Phenotype in Thyroid Cells

To investigate if the dedifferentiation effect of ER stress also involved alterations in the organization of the polarized epithelial monolayer, we analyzed CDH1 expression and distribution in PCCl3 cells.

**Figure 3:**
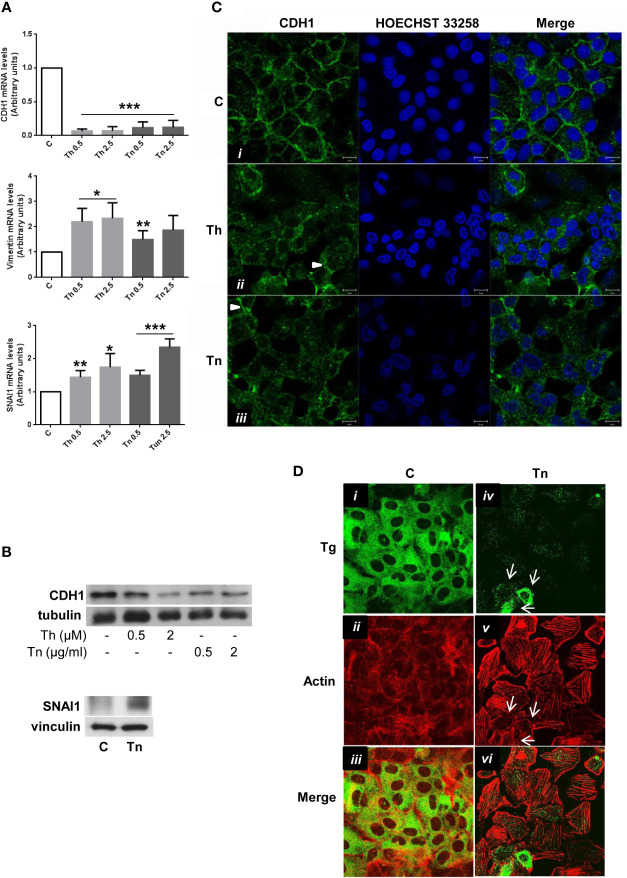
ER stress induces a shift towards a mesenchymal phenotype in PCCl3 cells. **(A)** Cells were plated in 100 mm diameter plates to about 80% confluence 24 h before treatments. Cells were treated or mock treated for 30 min with various concentrations of Th/Tn, followed by 24 h in complete medium without Th/Tn. Total RNA was extracted with the TRIzol reagent, according to the manufacturer’s protocol. Quantitative real-time RT-PCR analysis was performed as described in *Materials and Methods*. PCCl3 cells vehicle-treated (C) or treated with increasing concentrations of Th/Tn. *p < 0.05, **p < 0.01, ***p < 0.001, of each group respect to control. **(B)** Cells were plated in 60 mm diameter plates to about 80% confluence 24 h before treatments. Cells were treated or mock treated for 30 min with various concentrations of Th/Tn, followed by 24 h in complete medium without Th/Tn. Western blots of total protein extracts from PCCl3 cells vehicle-treated or treated with increasing concentrations of Th/Tn (CDH1) or with 0.5 μg/ml Tn (SNAI1). **(C)** PCCl3 cells were grown on glass coverslips for 48 h, then were vehicle-treated (*i*) or treated for 30 min with 0.5 μM Th or 0.5 μg/ml Tn (*ii*, *iii*, respectively). The medium was then replaced with medium without Th/Tn and cells incubated for 24 h. Cells were fixed in 4% paraformaldehyde in PBS for 20 min, washed twice in 50 mm NH_4_Cl in PBS, and permeabilized for 5 min in 0.1% Triton X-100 in PBS. Cells were double stained with anti-CDH1 antibodies and HOECHST 33258. Following Th/Tn treatments, the signal for CDH1 decreased. Arrowheads in (*ii*, *iii*) indicate residual CDH1 localized at the remaining cell-cell contacts. Bars, 10 μm. **(D)** PCCl3 cells were grown on glass coverslips for 48 h, then were vehicle-treated (*i*, *ii*, *iii*) or treated for 30 min with 0.5 μg/ml Tn (*iv*, *v*, *vi*). The medium was then replaced with medium without Tn and cells incubated for 24 h. Cells were fixed in 4% paraformaldehyde in PBS for 20 min, washed twice in 50 mm NH_4_Cl in PBS, and permeabilized for 5 min in 0.1% Triton X-100 in PBS. Cells were double-stained with anti-Tg antibodies and rhodamine-conjugated phalloidin. In control cells, rhodamine-conjugated phalloidin staining is mainly at the level of cortical actin. Following Tn treatment, the signal for Tg decreased and stress fibers were formed. Arrows indicate: few cells expressing various amounts of residual Tg (*iv*), the correlation between residual Tg expression and partially, not fully, formed stress fibers (*v*), and, consequently, the lack of overlap between Tg and actin signals (*vi*).

By real time RT-PCR and Western Blot, CDH1 was profoundly downregulated (**Figures 3A, B**, and **Supplementary Figures S6**, **S7**, **S9**). Next, we analyzed by immunofluorescence the cellular distribution of CDH1. In control conditions, CDH1 was mainly localized at cell-cell borders (**Figure 3C*i***). Following a treatment with Th/Tn, cells dramatically lost cell-cell contacts with residual CDH1 localized at the remaining contacts (arrowheads, **Figures 3C*ii*, C*iii***). Next, we studied the actin cytoskeleton organization and compared it with the distribution of a differentiation marker (Tg). In control cells, the Tg signal showed a distribution characteristic of ER, where it is co-translationally imported and folded (**Figure 3D*i***). The F-actin distribution was mainly cortical (**Figure 3D*ii***), as evidenced by Phalloidin staining, with the result that the signals of Tg and actin minimally overlapped (**Figure 3D*iii***). Following a Tn treatment, as expected, Tg abundance dramatically decreased, but a few cells still express small amounts of residual Tg, although they may be in the process to lose it (**Figure 3D*iv***, arrows). F-actin distribution profoundly changed with loss of cortical actin and formation of stress fibers (**Figure 3D*v***). In addition, in cells showing residual Tg expression there was also a decreased appearance of stress fibers (**Figure 3D*v***, arrows). Thus, Tg and actin signals remained distinct (**Figure 3D*vi***). These changes suggested a shift towards a mesenchymal phenotype, and therefore, we investigated the expression of mesenchymal markers.

Following Th/Tn treatments, vimentin mRNA increased (**Figure 3A**). Several transcription factors [snail1 (SNAI1) and snail2 (SNAI2), among others] downregulate transcriptionally CDH1 (28, 29). Thus, we found an increase of the mRNA and protein levels of SNAI1 following Th/Tn treatments (**Figures 3A, B**, and **Supplementary Figure S10**).

**Figure 4:**
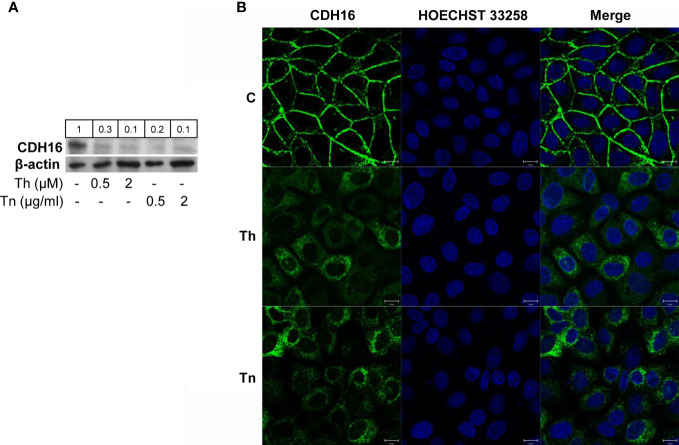
ER stress induces CDH16 downregulation in FRT cells. **(A)** Cells were plated in 60 mm diameter plates to about 80% confluence 24 h before treatments. Cells were treated or mock treated for 30 min with various concentrations of Th/Tn, followed by 24 h in complete medium without Th/Tn. Western blots of total protein extracts from FRT cells vehicle-treated or treated with increasing concentrations of Th/Tn. **(B)** FRT cells were grown on glass coverslips for 48 h, then were vehicle-treated (*i*) or treated for 30 min with 0.5 μM Th or 0.5 μg/ml Tn (*ii*, *iii*, respectively). The medium was then replaced with medium without Th/Tn and cells incubated for 24 h. Then, cells were fixed in 4% paraformaldehyde in PBS for 20 min, washed twice in 50 mm NH_4_Cl in PBS, and permeabilized for 5 min in 0.1% Triton X-100 in PBS. Cells were double stained with anti-CDH16 antibodies and HOECHST 33258. Following Th/Tn treatments, the signal for CDH16 dramatically decreased, and cell-cell contacts were lost. Bars, 10 μm.

Since PCCl3 cells express thyroid markers but display low level of cell polarity, we sought to extend our results to FRT cells that are well polarized both morphologically and functionally, although they are poorly differentiated (23). Remarkably, FRT cells, at variance with PCCl3 cells, express CDH16, the kidney-specific cadherin, also expressed in thyroid (26). CDH16 has been implicated in the differentiation of the kidney (30) and, recently, of the thyroid follicle (31). CDH16 was markedly downregulated after Th/Tn treatments (**Figure 4A**, and **Supplementary Figure S8**, **S9**). As for CDH1 in PCCl3 cells, we studied CDH16 cellular distribution in FRT cells. Under normal conditions, FRT cells showed very well-organized cell-cell junctions, with a strong CDH16 staining (**Figure 4B*i***). Yet, following Th/Tn treatments, CDH16 staining decreased and became intermittent and jagged, indicating, as for PCCl3 cells, dramatic alteration of cell-cell junctions (**Figures 4B*ii*, B*iii***). Thus, ER stress induced by Th/Tn caused, in both PCCl3 and FRT cells, similar detrimental changes in the cell-cell junction organization.

## Discussion

The accumulation of unfolded proteins in the lumen of ER induces a coordinated adaptive program called UPR. In metazoans, among other responses, the UPR upregulates transcriptionally genes that enhance the ER folding capacity and promote ERAD. If the adaptive response fails, cells execute apoptosis. While much attention has been devoted to the study of the survival/death switch (2–4), a new response to ER stress has emerged, which consists in an inhibition of differentiation (5–11).

In this study, we sought to extend the concept of regression to a less differentiated state following ER stress to tissue organization, focusing on an endocrine system. Thus, cellular dedifferentiation and shift towards a mesenchymal phenotype may be both present and part of a wide program of reshaping gene expression. To study these two different and perhaps complementary aspects, we decided to use the thyroid system, in which a highly cellular differentiation is coupled with a complex tissue organization, the thyroid follicle (19). Notably, in thyroid the differentiation genes (Tg, TPO, NIS) encode cargo proteins [like in pancreatic β-cell (insulin)] and in thyroid, genes involved in the organization of the follicle epithelium monolayer (cadherins) also encode cargo proteins (31, 32) [like in the structure of the pancreatic islet (33)]. Thus, in thyroid their down-regulation by ER stress would doubly impact on ER-specific protein load. Moreover, we decided to investigate another cytoprotective response to cellular stress, the induction of antioxidant enzymes (34, 35).

We used two thyroid cell lines, PCCl3 cells, in which both protein folding/misfolding (12–19) and differentiation (27) have been deeply studied at the molecular level, and FRT cells, which are highly polarized both at the structural and the functional level (23). Th/Tn disrupt the folding of Tg (13–19). Thus, Tg accumulates in the lumen of the ER, and the UPR is activated (14, and this study, **Figure 1**). The prevalent concept of the UPR outcome is dichotomous: cell survival, if the response restores a new equilibrium, or death, if the stress is severe and/or chronic and homeostasis cannot be restored (36). However, in recent years, another possibility has been described, inhibition of the dedifferentiated state. Thus, dedifferentiation has been shown in primary and immortalized chondrocytes following ER stress induced by Th/Tn (5), in hypertrophic chondrocytes of transgenic mice expressing a deletion mutant of collagen X (6), in lens fiber cells expressing mutant collagen IV (7), in hypertrophic chondrocytes of transgenic mice ectopically expressing a mutant Tg (cog Tg) driven by the collagen X promoter (37), and in a different line of thyroid cells, FRTL-5 cells (11). Interestingly, the misfolding of the same protein (Tg) in different cell type [ (11), this study, and (37)], and, conversely, the misfolding of different proteins (Tg and collagen X, 37 and 6, respectively) in the same cell type, causes an analogous outcome, dedifferentiation, which therefore appears to be neither protein- nor cell-specific.

However, following an ER stress, thyroid cells not only dedifferentiate, but also activate an antioxidant response (**Figure 2C**). Indeed, ER and oxidative stress are widely interconnected. ROS are produced while proteins fold in the lumen of the ER (38) but are overproduced in the presence of protein misfolding (39). Thus, ER stress (in our case produced by Th/Tn) causes oxidative stress (39). In turn, oxidative stress exacerbates ER stress, since ROS inactivate SERCA 3 and 2b pumps (40), causing a Ca2+ loss from the ER lumen and protein misfolding. Both SERCAs are expressed in thyroid, with a prevalence of SERCA 2b (41). Given this vicious cycle, a cellular response, to be effectively cytoprotective in the short term, has to counteract both protein misfolding and ROS accumulation. It is what we have observed in PCCl3 cells, with the upregulation of both, molecular chaperones and antioxidant enzymes following an ER stress.

Furthermore, we report ER stress negatively impact on epithelial tissue organization. Indeed, we show that expression and localization of CDH1 and CDH16 is dramatically altered following ER stress in PCCl3 and FRT cells, respectively. In PCCl3 cells expression of vimentin increases, while the actin cytoskeleton is reorganized with formation of stress fibers. These results may be explained, at least in part, by the induction of SNAI1, known to repress CDH1 transcription (28), to induce vimentin expression (42), to cause disappearance of cortical actin and formation of stress fibers (43) (see **Figure 3D**), and, more in general, to induce EMT (42–44).

Strikingly, disappearance of cortical actin and formation of stress fibers co-exist with downregulation of thyroid markers in the same cell (**Figure 3D**). Even more strikingly, in cells where the loss of Tg expression was marginal, the actin re-organization (disappearance of cortical actin and formation of stress fibers) was less evident (**Figure 3D**, arrows), suggesting a possible causal link between these two phenomena. That a link between dedifferentiation and mesenchymal shift may exist is suggested also by two studies (5, 45). Yang et al. (5) reported that ER stress induces downregulation of mRNAs of differentiation markers of prehypertrophic chondrocytes, while Seki et al. (45) reported that SNAI1 inhibits transcription of these markers by binding to promoter E-boxes, during the chondrocyte prehypertrophic to the hypertrophic passage. Thus, the prehypertrophic-hypertrophic passage may impose ER stress on chondrocytes (in a way similar to lymphocyte to plasma cell transition) (46), and the resulting upregulation of SNAI1 links dedifferentiation to EMT. A similar mechanism may be present in thyroid. SNAI1, upregulated by ER stress, may inhibit thyroid differentiation repressing transcription of thyroid transcription factors. These results confirm the conclusions of **Figures 1**, **2**, **4** of the paper by the same authors that were object of concerns causing the retraction of the paper (47). These results also extend previous conclusions, by showing that also the thyroid (and kidney)-specific cadherin-16 was downregulated and antioxidant genes were upregulated following ER stress. The new finding concerning CDH16 is of particular interest in light of the recent demonstration that this thyroid-specific cadherin controls apical-basal follicular polarization and follicle formation (31).

In conclusion, our results describe a new strategy, besides survival or death, in the cell response to ER stress. Thus, following ER stress, thyroid cells execute an antioxidant response and regress to a less differentiated state, not only involving tissue-specific proteins, but also epithelial tissue differentiation and organization, shifting towards a mesenchymal phenotype. These results highlight, at the same time, a new and wide strategy to achieve survival following ER stress, but also, in a sort of the other side of the coin, a possible new molecular mechanism of decline/loss of function leading to a deficit of thyroid hormones formation.

## Data Availability Statement

The raw data supporting the conclusions of this article will be made available by the authors, without undue reservation.

## Author Contributions

BJ conceived the biological problem underlying the manuscript. LU, PM, CG, GC, and BJ designed the experiments and analyzed the results. LU, PM, CG, GC, DC, AT, AM, DP, and GR performed the experiments. LU, PM, CG, GC, CM, FB, EC, and BJ discussed during the course of the experimental work. BJ wrote the paper. All authors contributed to the article and approved the submitted version.

## Funding

This research was funded, in part, by the Ministero dell′Istruzione, Università e della Ricerca Scientifica (grants PRIN 2017 and PON “RICERCA E INNOVAZIONE” 2014–2020 E FSC-progetto “Innovative Devices For SHAping the RIsk of Diabetes” (IDF SHARID)-ARS01_01270), by the Regione Campania (POR FESR 2014–2020—Obiettivo specifico 1.2.—Manifestazione di Interesse per la Realizzazione di Technology Platform nell′ambito della Lotta alle Patologie Oncologiche”—Projects COEPICA, RARE PLAT NET and SATIN), by the Consiglio Nazionale delle Ricerche (grant FLAGSHIP Interomics Project ASPIRE), by the Italian Diabete Ricerca Foundation and Eli Lilly Italy (2018–2020), and by the European Foundation for the Study of Diabetes (EFSD)/Boehringer Ingelheim (2018–2020).

## Conflict of Interest

The authors declare that the research was conducted in the absence of any commercial or financial relationships that could be construed as a potential conflict of interest.

## Abbreviations

ATF4, activating transcription factor-4; ATF6, activating transcription factor-6; CDH1, cadherin-1; CDH16, thyroid (and kidney)-specific cadherin-16; eIF2α, eukaryotic translation initiation factor 2 alpha; EMT, epithelial-mesenchymal transition; ER, endoplasmic reticulum; ERAD, endoplasmic reticulum-associated degradation; GAPDH, Glyceraldehyde-3-Phosphate Dehydrogenase; GRP78, glucose-regulated protein 78; HO1, heme oxigenase 1; NIS, sodium-iodide symporter; Pax-8, paired box gene 8; SNAI1, snail 1; SNAI2, snail 2; SOD1, superoxide dismutase 1; Tg, thyroglobulin; Th/Tn, thapsigargin and tunicamycin; TTF-1, thyroid transcription factor 1; TPO, thyroperoxidase; TXNRD1, thioredoxin reductase 1; UPR, unfolded protein response; XBP-1s, spliced active form of X-box binding protein-1.
