# Supplementary figures and images for: The Pervasive Effects of ER Stress on a Typical Endocrine Cell: Dedifferentiation, Mesenchymal Shift and Antioxidant Response in the Thyrocyte
Source: Front Endocrinol (Lausanne). 2020 Nov 9;11:588685. doi: 10.3389/fendo.2020.588685 (PMC7680880; doi:10.3389/fendo.2020.588685)

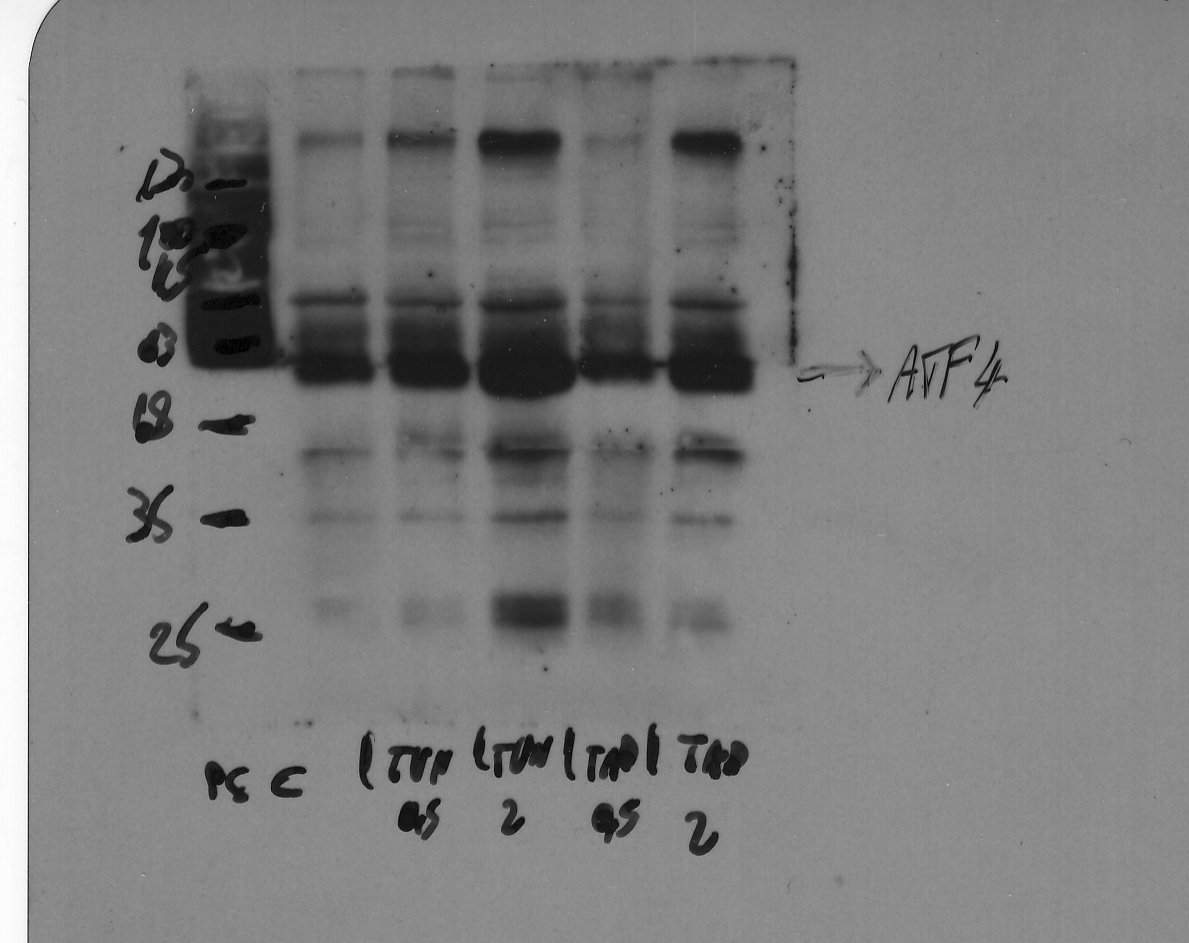

Supplement: Supplementary file 1 [file Image_1.jpeg]

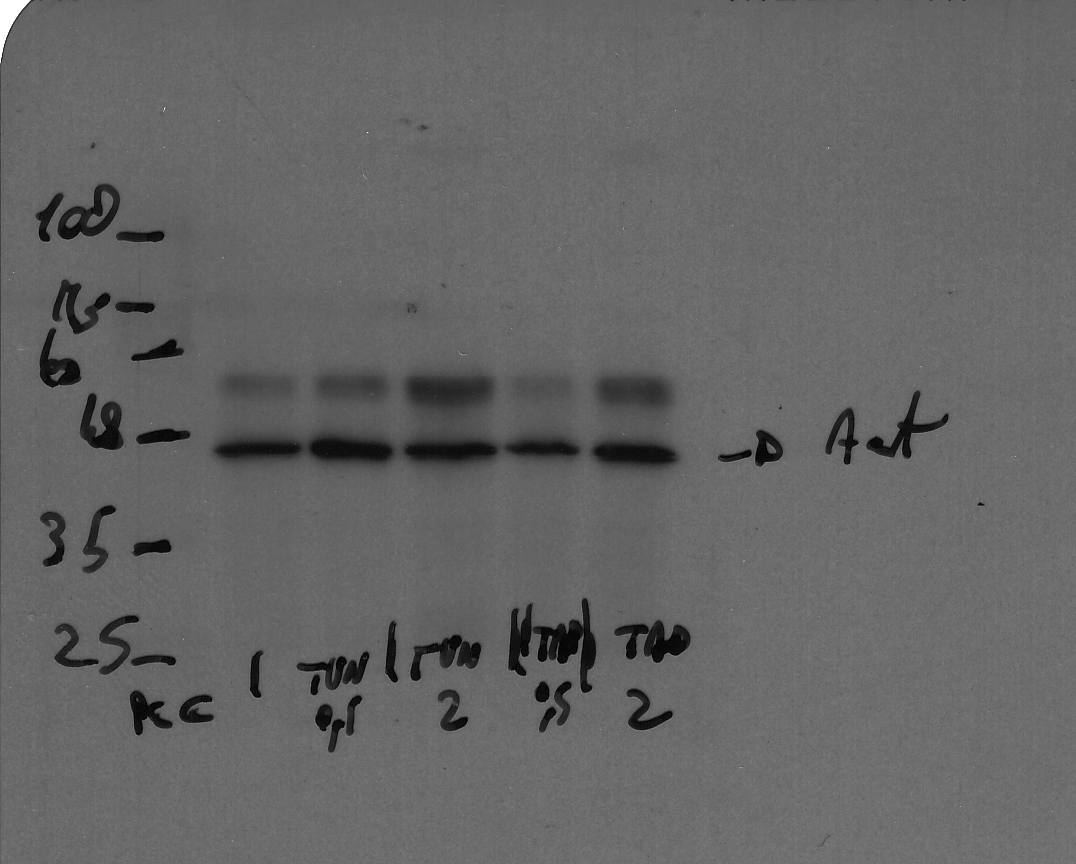

Supplement: Supplementary file 2 [file Image_2.jpeg]

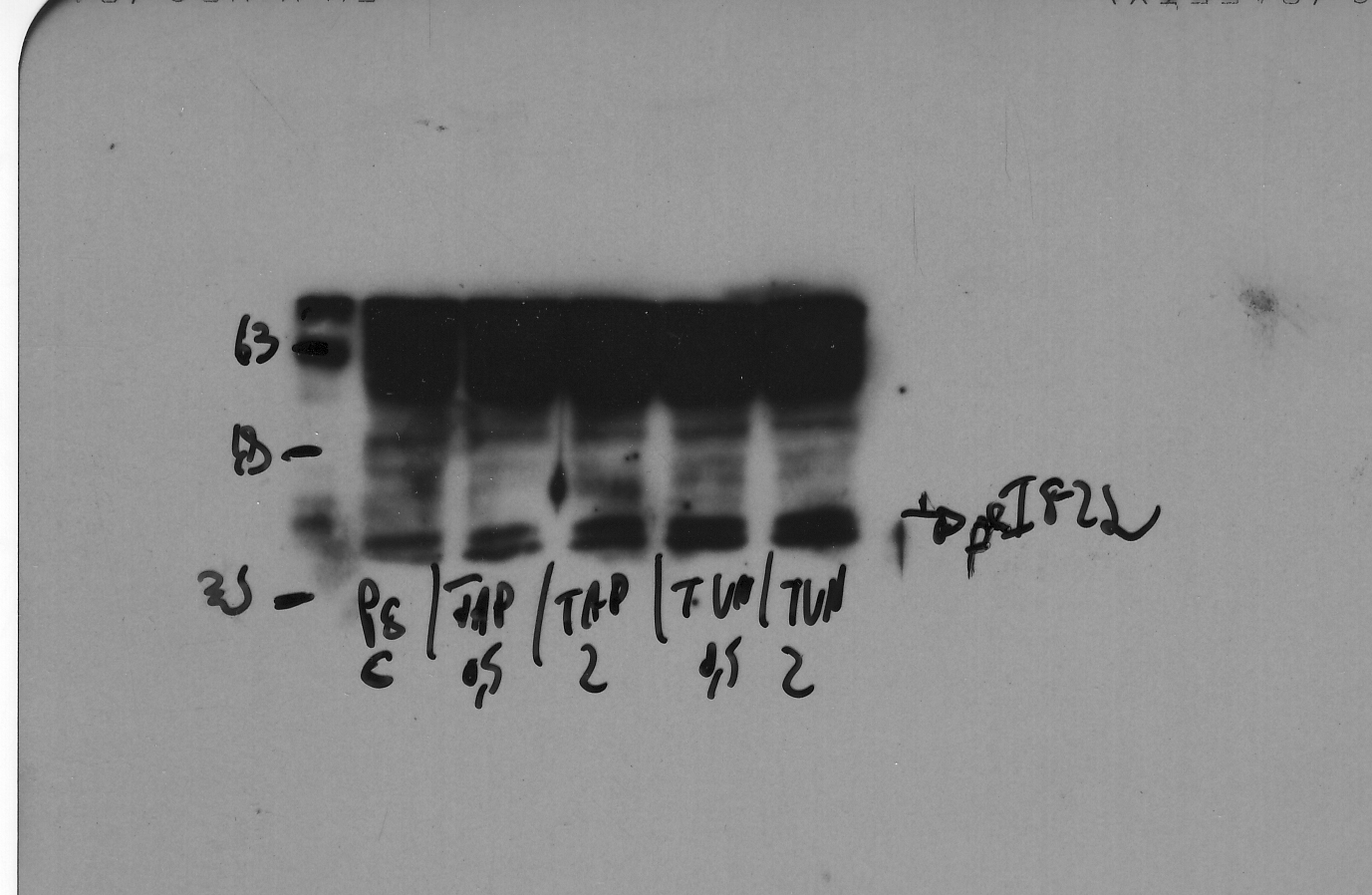

Supplement: Supplementary file 3 [file Image_3.jpeg]

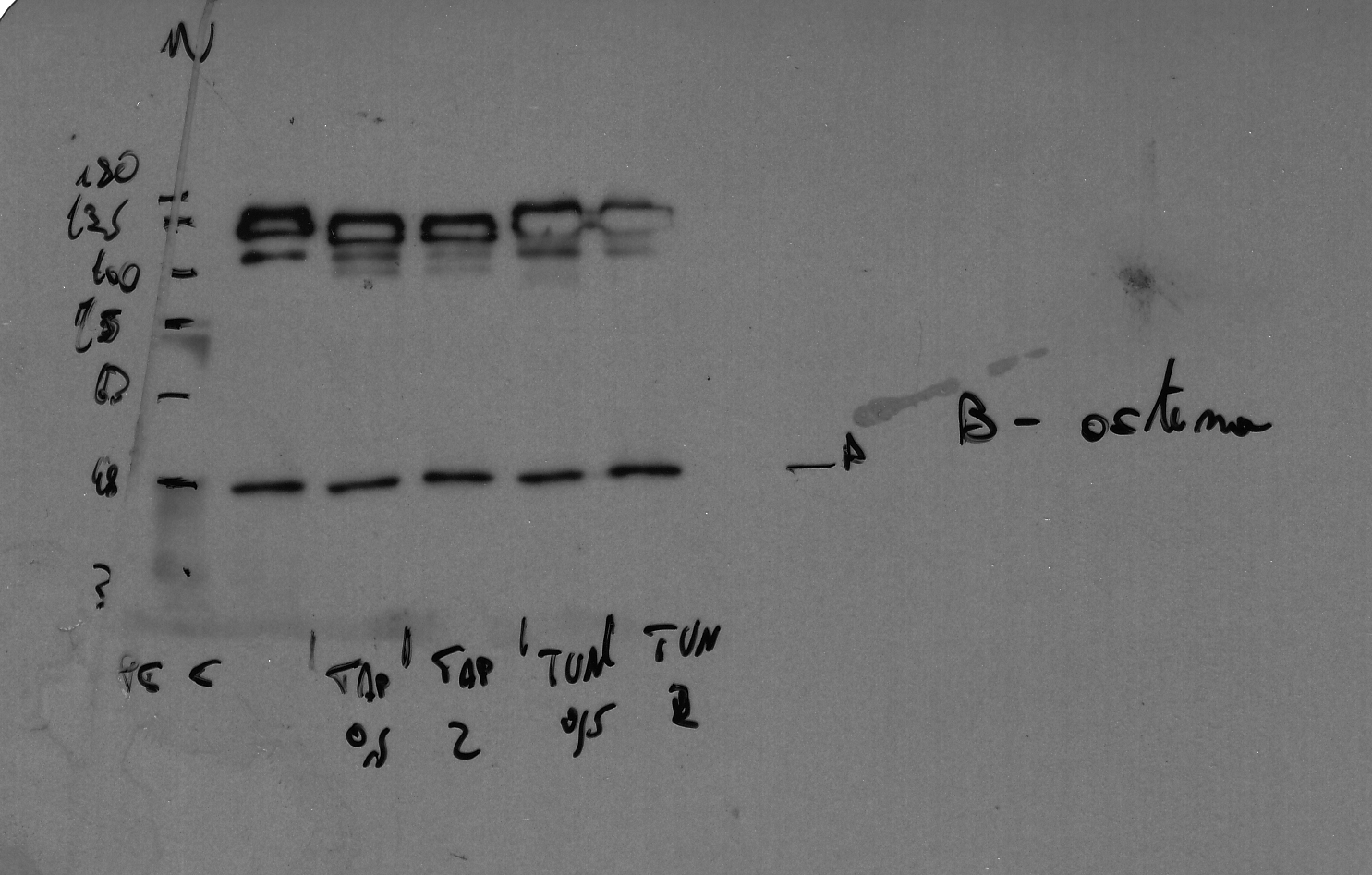

Supplement: Supplementary file 4 [file Image_4.jpeg]

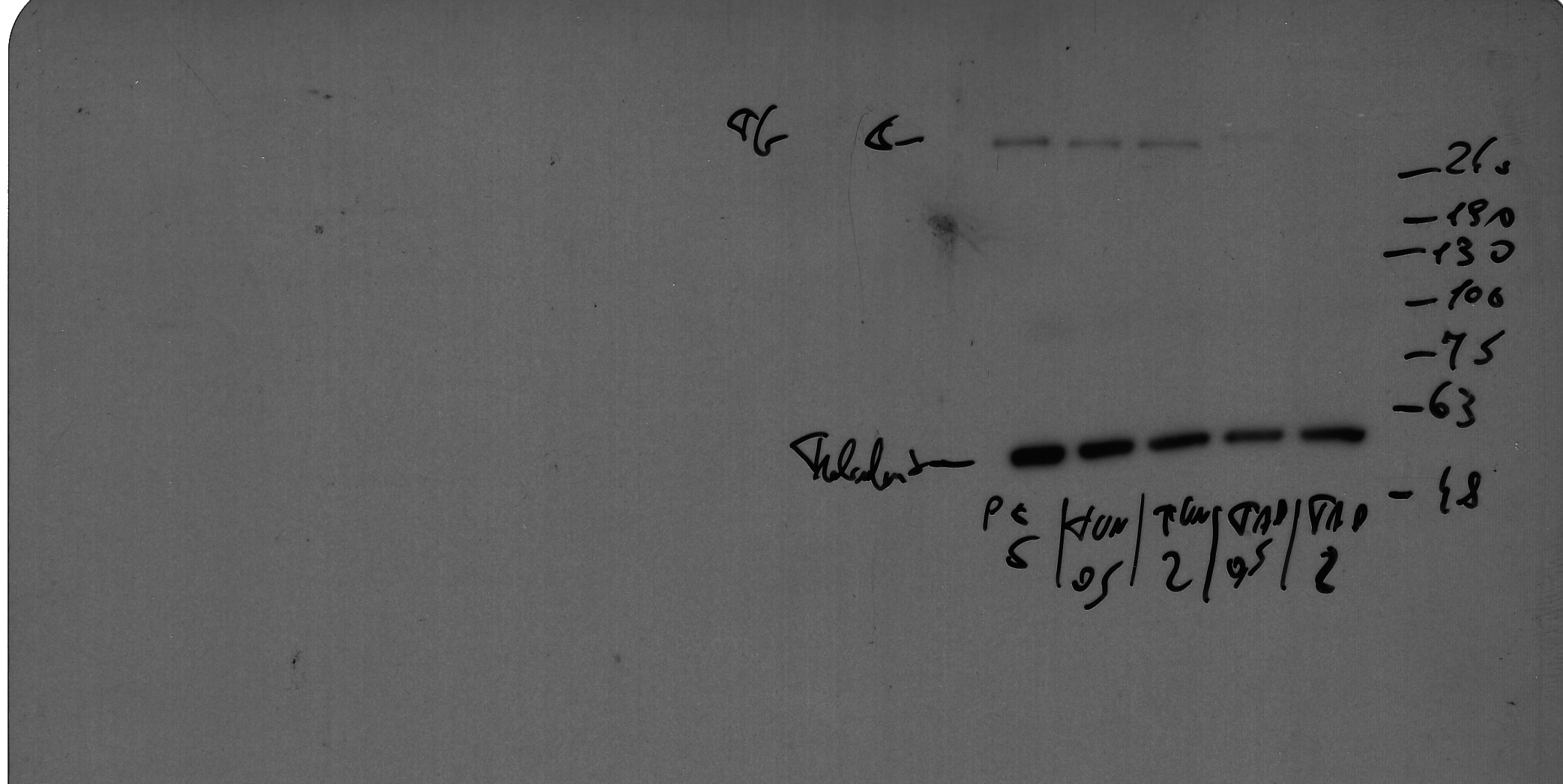

Supplement: Supplementary file 5 [file Image_5.jpeg]

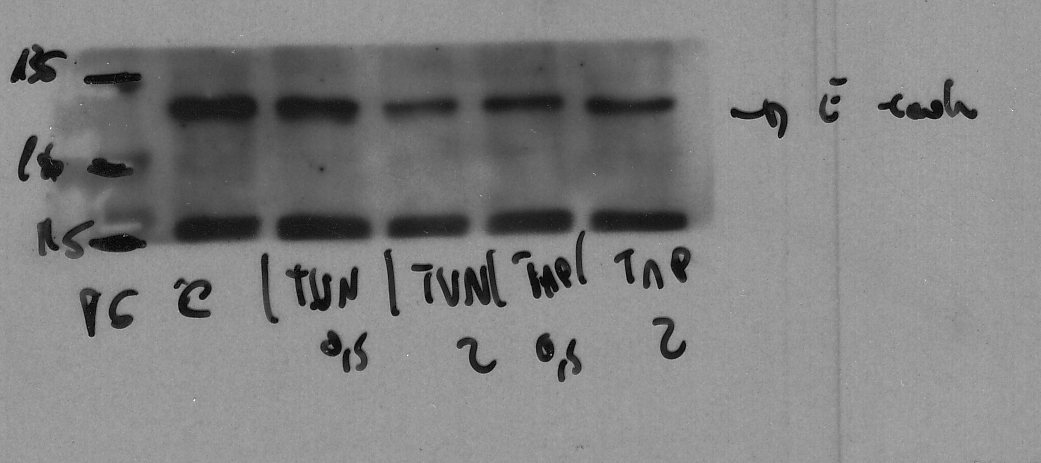

Supplement: Supplementary file 6 [file Image_6.jpeg]

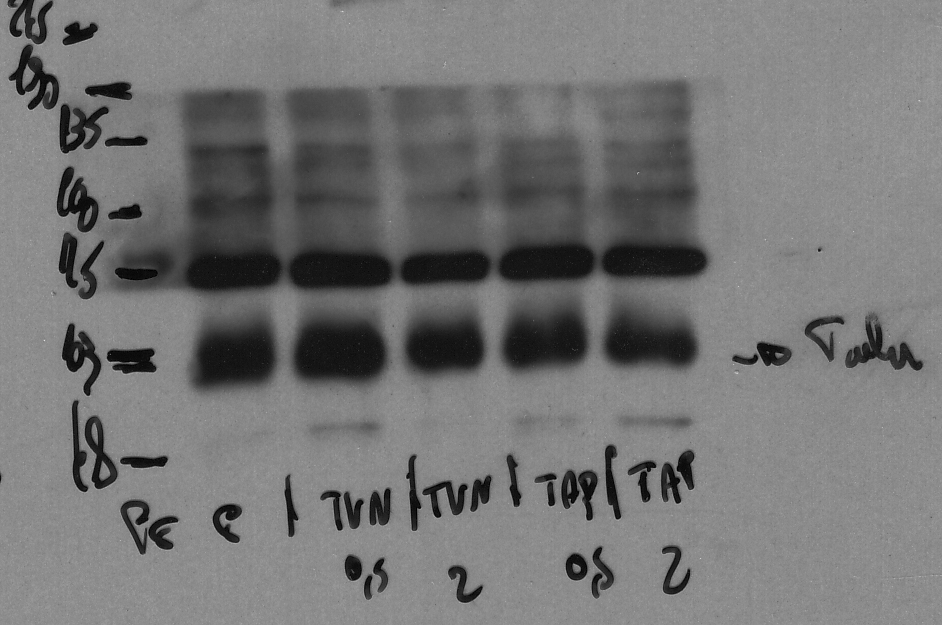

Supplement: Supplementary file 7 [file Image_7.jpeg]

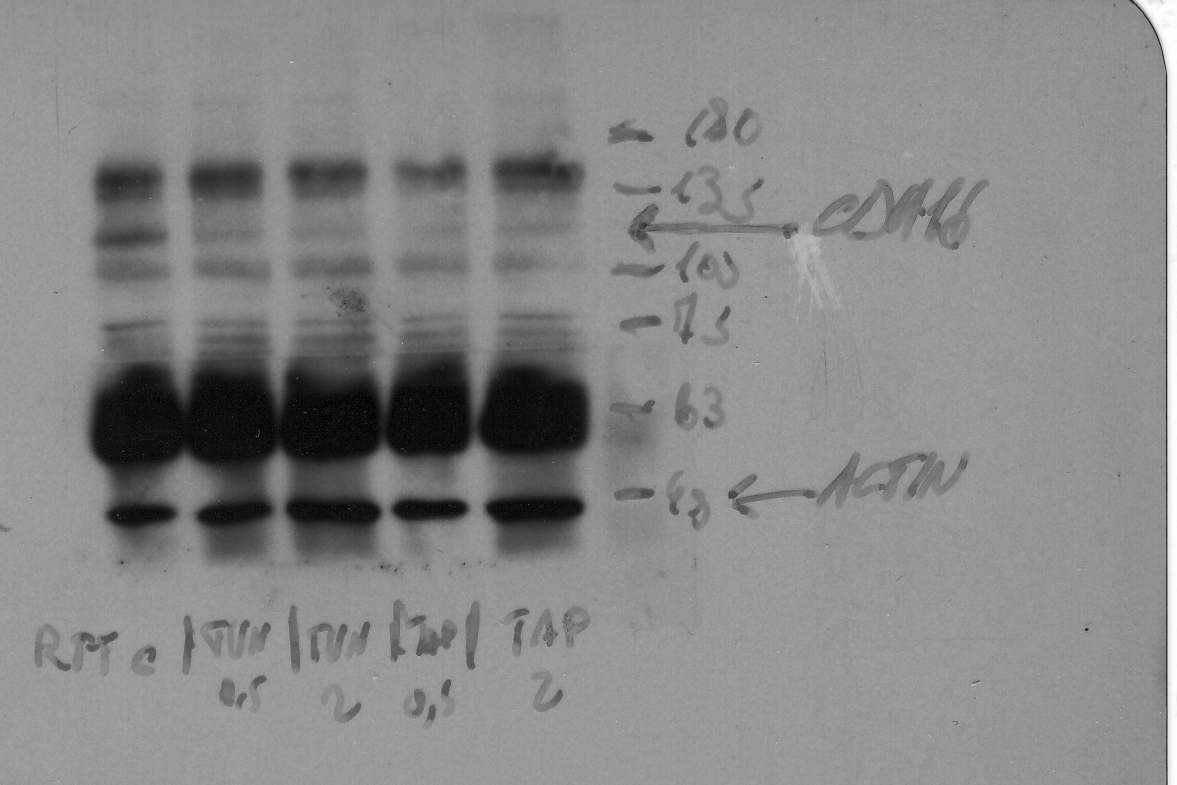

Supplement: Supplementary file 8 [file Image_8.jpeg]

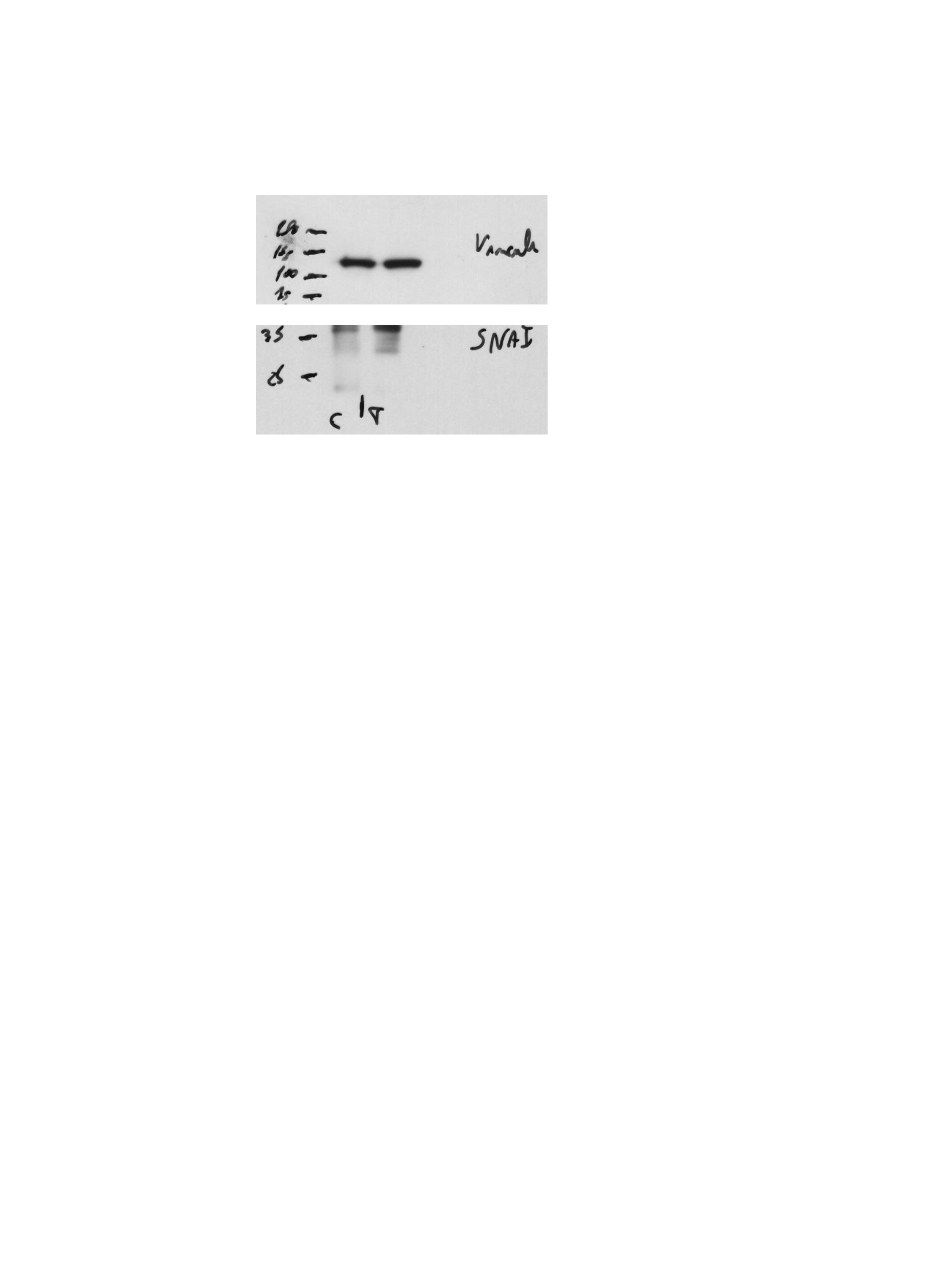

Supplement: Supplementary file 9 [file Image_9.jpeg]
