# Supplementary material for: The Pervasive Effects of ER Stress on a Typical Endocrine Cell: Dedifferentiation, Mesenchymal Shift and Antioxidant Response in the Thyrocyte
Source: Front Endocrinol (Lausanne). 2020 Nov 9;11:588685. doi: 10.3389/fendo.2020.588685 (PMC7680880; doi:10.3389/fendo.2020.588685)
Supplement: Supplementary file 10 [file Table_1.doc]

**SUPPLEMENTARY MATERIALS and METHODS.**

#### Primers used for Real-time Reverse Transcription-Polymerase Chain Reaction (RT-PCR).

ATF4: 5’-CTATCTGGAGGTGGCCAAGC-3’ and 5’-TCTGTCCCGGAAAAGGCATC-3’

ATF6: 5’-GACTGGGAGTCCACGTTGTT-3’ and 5’-TGTCTGACTCCCAAGGCATC-3’

CDH1: 5’-CCCGGAAAATCAAAAGGGC-3’ and 5’-GATGAAAACGCCAACAGGG-3’

GAPDH: 5’-GCCTTCCGTGTTCCTACC-3’ and 5’-AGAGTGGGAGTTGCTGTT-3’

GRP78: 5’-AAGCCCGTCCAGAAAGTGTT-3’ and 5’-ATCTGGGTTTATGCCACGGG-3’

NIS: 5’-AAGTGACCGGGTTGGACATC-3’ and 5’-AGCCAACGAGCATTACCACA-3’

Pax8: 5’-GGACAGTTGTCGACTGAGCA-3’ and 5’-GAATGAGGATCTGCCACCAC-3’

SNAI1: 5’-ACCCTCATCTGGGACTCTCT-3’ and 5’-CTGGGAGCTTTTGCCACTGT-3’

sXBP1: 5’-TGCTGAGTCCGCAGCAGGTG-3’ and 5’-GCTGGCAGGCTCTGGGGAAG-3’

Tg: 5’-GGCCTCCACCTTCACTCAAA-3’ and 5’-ACCAAAGCTGGTGGCAGTAA-3’

TPO: 5’-CCTCAAACTCCTCACGGACC-3’ and 5’-AAGCCATTCATCTGCTGCCT-3’

TTF-1: 5’-CGATGAGTCCAAAGCACACG-3’ and 5’-CTGGCCCTGTCTGTAAGCTG-3’

Vimentin: 5’-TGAGATCGCCACCTACAGGA-3’ and 5’-GGAGTGGGTGTCAACCAGAG-3’

TXNRD1: 5’-TGGGTCCAAATGCTGGAGAG-3’ and 5’-CTCCCCCAGAACGCTTAGTC-3’

SOD1: 5’-GAGCATTCCATCATTGGCCG-3’and 5’-GGCAATCCCAATCACACCAC-3’

HO-1: 5’-GTCCCAGGATTTGTCCGAGG-3’ and 5’-GTACAAGGAGGCCATCACCA-3’
