# Supplementary material for: The Pervasive Effects of ER Stress on a Typical Endocrine Cell: Dedifferentiation, Mesenchymal Shift and Antioxidant Response in the Thyrocyte
Source: Front Endocrinol (Lausanne). 2020 Nov 9;11:588685. doi: 10.3389/fendo.2020.588685 (PMC7680880; doi:10.3389/fendo.2020.588685)
Supplement: Supplementary file 11 [file Table_2.doc]

Legends to Supplementary Material Figures S9.

Fig. S1. ATF4 western blots of total protein extracts from PCCl3 cells vehicle-treated or treated with increasing concentrations of Th/Tn. Molecular mass markers are: 130, 100, 75, 63,48,35, and 25 kDa.

Fig. S2: β-actin western blots on the same filter of Fig. S1. Molecular mass markers are:100, 75, 63,48,35, and 25 kDa.

Fig. S3: p-eIF2α western blots of total protein extracts from PCCl3 cells vehicle-treated or treated with increasing concentrations of Th/Tn. Molecular mass markers are: 63, 48, and 35 kDa.

Fig. S4: β-actin western blots on the same filter of Fig. S3. Molecular mass markers are:180, 135, 100, 75, 63, 48, and 35 kDa.

Fig. S5: Tg and β-actin western blots of total protein extracts from PCCl3 cells vehicle-treated or treated with increasing concentrations of Th/Tn. Molecular mass markers are: 260, 180, 130, 100, 75, 63, and 48 kDa.

Fig. S6: CDH1 western blots of total protein extracts from PCCl3 cells vehicle-treated or treated with increasing concentrations of Th/Tn. Molecular mass markers are: 135, 100, and 75 kDa.

Fig. S7: tubulin western blots on the same filter of Fig. S6. Molecular mass markers are:265, 180, 135, 100, 75, 63, and 48 kDa.

Fig. S8: CDH16 and β-actin western blots of total protein extracts from PCCl3 cells vehicle-treated or treated with increasing concentrations of Th/Tn. Molecular mass markers are: 180, 135, 100, 75, 63, and 48 kDa.
